# Supplementary material for: Eating Occasions, Obesity and Related Behaviors in Working Adults: Does it Matter When You Snack?
Source: Nutrients. 2019 Oct 1;11(10):2320. doi: 10.3390/nu11102320 (PMC6835708; doi:10.3390/nu11102320)
Supplement: Supplementary file 1 [file nutrients-11-02320-s001.pdf]

Supplementary Material

24. Describe how you eat during a **typical 24-hour** period. Record the time for each eating episode and mark with an **X** the type of meal which corresponds best.

Example

Note: You can have several main meals during a day.
Do not forget snack, other 'light meals', and drinks.

Time	Type of Meal			
	**Main Meal** (e.g., cooked dish, hearty soup with bread, Chef salad with bread, pizza)	**Light Meal/Breakfast** (e.g., cooked or cold cereals, simple soup, side salad)	**Snack Meal** (e.g., cookie, slice of cake/pie, energy bar, chips, fruit, candy, ice cream – w/ or w/o a drink)	**Drink, only** (e.g., coffee, tea, soft drink, juice, milk beer, wine)
6:30am				x
10:00 am			x	
12:00 pm	x			
3:00 pm		x		
6:00 pm	x			
9:00 pm			x	

(*Please fill out table below.*)

Time	Type of Meal			
	**Main Meal** (*e.g., cooked dish, hearty soup with bread, Chef salad with bread, pizza*)	**Light Meal/Breakfast** (*e.g., cooked or cold cereals, simple soup, side salad*)	**Snack Meal** (*e.g., cookie, slice of cake/pie, energy bar, chips, fruit, candy, ice cream – w/ or w/o a drink*)	**Drink, only** (*e.g., coffee, tea, soft drink, juice, milk beer, wine*)

Figure S1. Adaptation of eating pattern assessment[1] used in the Promoting Activity and Changes in Eating (PACE) study, 2005–2009.

1. Berteus Forslund H, Lindroos AK, Sjostrom L, Lissner L. Meal patterns and obesity in Swedish women-a simple instrument describing usual meal types, frequency and temporal distribution. *European journal of clinical nutrition.* 2002;56(8):740-747.
